# *NUF2* Expression Promotes Lung Adenocarcinoma Progression and Is Associated With Poor Prognosis

**DOI:** 10.3389/fonc.2022.795971

**Published:** 2022-06-23

**Authors:** Feng Jiang, Xiaolu Huang, Xiang Yang, Huixin Zhou, Yumin Wang

**Affiliations:** ^1^ Department of Laboratory Medicine, The First Affiliated Hospital of Wenzhou Medical University, Wenzhou, China; ^2^ Key Laboratory of Clinical Laboratory Diagnosis and Translational Research of Zhejiang Province, Wenzhou, China

**Keywords:** NUF2, prognosis, immune infiltration, lung adenocarcinoma, survival

## Abstract

Aberrant expression of the gene encoding the Ndc80 kinetochore complex component (*NUF2*) reportedly contributes to the progression of several human cancers. However, the functional roles of *NUF2* and their underlying mechanisms in lung adenocarcinoma (LUAD) are largely unknown. The current study aimed to investigate the role of *NUF2* in LUAD tumorigenesis. Here, TCGA, ONCOMINE, the Human Protein Atlas, UALCAN, and the results of our cohort were used to analyze the expression of *NUF2* in LUAD. A Kaplan–Meier analysis and univariate and multivariate Cox regression analyses were performed to estimate the prognostic values of *NUF2* expression in the Cancer Genome Atlas cohort. We studied the effects of *NUF2* expression on proliferation, migration, invasion, and tumor growth using LUAD cell lines. Gene set enrichment analysis (GSEA) was used to analyze the pathways and biological function enrichment of *NUF2* in LUAD. The ssGSEA database was used to analyze the relationship between *NUF2* expression and immune cell infiltration in LUAD. Results revealed elevated expression of *NUF2* in LUAD specimens. Patients overexpressing *NUF2* had poor prognoses relative to those with low *NUF2* expression. Knockdown of *NUF2* suppressed the proliferation, migration, invasion, epithelial-mesenchymal transition, and colony formation of LUAD cells. Moreover, *NUF2* knockdown induced cell cycle arrest at the G0/G1 phase. Gene Ontology and GSEA analyses suggested that *NUF2* may be involved in immunity, proliferation, and apoptosis-related pathways. *NUF2* overexpression was positively correlated with differential immune cell infiltration. In conclusion, *NUF2* expression was associated with the clinical phenotype of LUAD and hence has potential implications in LUAD treatment.

## Introduction

Lung adenocarcinoma (LUAD) is the most prevalent subtype of non-small cell lung cancer (NSCLC) (1). Since its early symptoms are not obvious, LUAD is mostly diagnosed in advanced stages (2). Thus, reliable biomarkers to estimate the key parameters for diagnosis and disease monitoring are urgently needed.

The NDC80 complex, which is composed of NDC80 (also known as HEC1), NUF2, SPC24, and SPC25, is the primary microtubule receptor at the kinetochore. *NUF2* is a key regulator of the cell cycle and is overexpressed in several types of cancers, including bladder cancer, renal cell carcinoma, cholangiocarcinoma, and lung cancer (3). Mounting evidence suggests that *NUF2* is a valuable prognostic biomarker for detecting breast cancer, hepatocellular carcinoma, and oral cancer (4–6). *NUF2*, as a part of the aforementioned NDC80 complex, is differentially expressed in tumor tissues and has diagnostic value for LUAD (7). Moreover, this gene combination has a better diagnostic value for LUAD than *NUF2* alone. *NUF2* overexpression is reportedly associated with poor overall patient survival. Reports have also suggested that *NUF2* is a potential prognostic biomarker in NSCLC (8). Taken together, *NUF2* appears to play an important role in carcinogenesis, although systematic studies are required to further shed light on its functional and clinical role as a biomarker for LUAD.

Immunotherapy has generated significant interest in the context of NSCLC treatment, especially with the recent success of immune checkpoint inhibitors for treating metastatic stage IV cancers (9, 10). Recently, focus has shifted towards novel strategies that target *LAG3* and *TIM3*, as well as targeting Tregs and their immunosuppressive factors (e.g. TGF-β) into the tumor microenvironment, which have been proposed as more effective in stimulating an anti-tumor immune response (11). The tumor microenvironment is an active research field for tumor diagnosis, treatment targets, and prognostic biomarkers (12). Overall, the study of immune-related therapeutic targets is essential for effective treatment of lung cancer.

In this study, we evaluated the expression of *NUF2* in LUAD as described in the following five cohorts: The Cancer Genome Atlas (TCGA), Genotype-Tissue Expression (GTEx), Gene Expression Omnibus (GEO), ONCOMINE, and UALCAN. Furthermore, we used our independent cohort to identify the expression pattern of *NUF2*, as well as the related clinicopathological features. We used TCGA to explore the role of *NUF2* expression level in LUAD as a pathological and prognostic biomarker. A nomogram integrating clinicopathological indexes and *NUF2* expression was established to predict prognosis. Moreover, we examined the association between *NUF2* expression and immune cell infiltration. We used *NUF2-*targeting siRNA to suppress its endogenous expression to examine its role in LUAD. The relationships between *NUF2* and its pathways were analyzed using gene set expression analysis (GSEA).

## Materials and Methods

### Patient and Sample

Specimens of LUAD tissues and para-cancerous tissues were obtained from 61 patients with LUAD, who had undergone surgical operation in the First Affiliated Hospital of Wenzhou Medical University, China, from February 2017 to March 2020. The inclusion criteria for patients were as follows: (I) confirmed histology following pathology review; (II) aged between 18 and 80 years; (III) absence of palliative surgery or neoadjuvant chemo- and/or radiotherapy; (IV) no major organ dysfunction unless caused by the malignant disease; and (V) no history of major neurological or psychiatric disease; (IV) no significant renal dysfunction, cardiovascular or cerebrovascular diseases, hematological or endocrine system diseases, or metabolic illness; and (III) psychiatric illness.

### Data Mining

In the first round of verification of *NUF2* expression, genomic data of samples from subjects with LUAD (n = 513) and from 59 matched normal lungs were obtained from TCGA-LUAD; 57 paired LUAD and adjacent normal tissue samples were included in TCGA. To evaluate *NUF2* expression, tumor tissues were obtained from TCGA, and normal tissues from the TCGA and GTEx databases were combined. Thereafter, we searched the GEO database and downloaded human LUAD-related datasets. Among them, 57 LUAD patients and 11 normal specimens were included in GSE116959, and 52 pairs of LUAD and normal lung specimens were included in GSE115002. To investigate *NUF2* expression in other databases, we referred to the ONCOMINE database (https://www.oncomine.org/resource/main.html).

In the second round of verification, differential expression of the NUF2 protein between normal human lung and LUAD tissues was determined using the UALCAN database (http://ualcan.path.uab.edu/) using 111 LUAD and 111 healthy lung tissues.

In the third round of experimental verification, 61 pairs of LUAD tissue and paired tissue samples stored in our laboratory were used. *NUF2* mRNA transcription levels were evaluated in tissue and cancer cell lines (HCC827, A549, and SPCA1). Briefly, 500 ng total RNA (for each sample) was reverse-transcribed using the first-strand cDNA synthesis kit (TaKaRa, Kusatsu, Japan) in accordance with the manufacturer’s instructions. cDNAs were then used for RT-qPCR analysis using SYBR Green (TaKaRa). The primers used were as follows: NUF2 forward primer, 5′-TTTTGCCTATCTGCCGGGTG-3′; reverse, 5′-GTCCGCAGAGGATTTATATTGCC-3′. β-actin forward primer: 5′-CCTGGCACCCAGCACAAT-3′; reverse primer, 5′-GCTGATCCACATCTGCTGGAA-3′.

### Nomogram Construction and Validation

Next, a nomogram integrating clinical prognostic factors, such as primary therapeutic outcome, tumor status, pathologic stage, an *NUF2* expression was constructed.The *NUF2* expression profile was used to predict 1-, 3-, and 5-year prognoses of patients with LUAD with the R rms package. Calibration curves were established to examine the accuracy of the nomogram. Finally, C-index was used to evaluate the predictive ability of the model.

### Functional Analyses

The associations between *NUF2* expression and biological processes, molecular functions, and cellular components were evaluated by Gene Ontology (GO) annotation. The impact of *NUF2* on pathway activation and inhibition was evaluated using GSEA, a web service aimed at unmasking potential cancer-related pathways.

### Protein-Protein Interaction Network Analysis

STRING (https://string-db.org/), a tool for predicting protein-protein interactions (PPIs), was used to quantify the functional interactions of the NUF2 protein (13). A combined score > 0.4 was set as the cut-off.

### Estimation of Relative Abundance of Immune Cell Types

Single-sample gene set enrichment analysis (ssGSEA) is a database that shows the relative abundance of immune cells in LUAD. ssGSEA was used to evaluate the infiltration level of 22 types of immune cells in LUAD samples based on their *NUF2* expression profile. Subsequently, differences in the composition of immune cells between low-risk and high-risk patients with *NUF2* expression was compared.

### Immunohistochemical Staining of the Tissue Microarray

Samples from 7 LUAD patients were stained with rabbit polyclonal antibodies against NUF2 (1:500, #ab244470, Abcam). The staining results were evaluated by two independent pathologists. Staining intensity was classified as 0 (no staining), 1 (weak staining), 2 (moderate staining), and 3 (strong staining). The amounts of positive tumor cells were classified according to the following percentages: 1+ (≤25%), 2+ (26%–50%), 3+ (51%–75%), and 4+ (>75%). The final expression scores were calculated by multiplying the two variables together.

### Cell Line and siRNA Transfection

LUAD cell lines HCC827 and A549 were used to determine the regulation of *NUF2* expression. siRNAs targeting NUF2 were transfected into HCC827 and A549 cells using Polyethylenimine (PEI); briefly, 35 × 10^4^ cells/well were seeded in 6-well plates, and incubated with 50 nM siRNA and 4 μg/ml PEI. After 8 h, the medium was replaced with complete medium.

### Cell Proliferation Assay

A549 and HCC827 cells and Cell Counting Kit-8 (CCK-8) kit (TaKaRa) were used for the CCK-8 assay. A total of 1500 LUAD cells in full medium were seeded in 96-well plates. The next day, transfections were performed with PEI (4 μg/ml) for 8h. Briefly, the 96-well plates containing the transfected cells were incubated at 37 °C for the indicated time points. CCK-8 reaction solution (10%) was added to the cells and incubated for 1 h at 37°C. The optical density (OD) values at 450 nm were measured with a microplate reader (Thermo Fisher, Waltham, MA, USA) to analyze the number of proliferating cells.

### Colony Formation Assay

A549 and HCC827 cells (600 cells/well), transfected with siRNAs, were seeded in 6-well plates. Subsequently, the plates were incubated under standard culture conditions for 14 days and stained with 0.1% crystal violet (Beyotime, Shanghai, China) for 1 h. Finally, cells were imaged under a microscope (Olympus, Tokyo, Japan).

### Cell Cycle Analysis

A549 and HCC827 cells were seeded in 6-well plates at 2.0 × 10^5^ cells per well and subjected to various transfections. Thereafter, the cells were harvested and fixed in 75% (v/v) ethanol overnight at −20°C. Subsequently, the cells were resuspended in cold PBS and incubated in the dark for 30 min at room temperature in a buffer containing 25 mg/mL 7-aminoactinomycin (7-AAD Sigma Aldrich, St. Louis, MO, USA) and 40 mg/mL RNase. Subsequently, the cells were analyzed by flow cytometry (FACS Calibur, BD Biosciences, Franklin Lakes, NJ, USA), and the percentage of cells in different phases of the cell cycle was determined.

### Transwell Assay

Two days after transfection, A549 and HCC827 cells were plated at a density of 2.5 × 10^5^ in the upper chamber of the transwell plate (8 μm, Corning, Tewksbury, MA, USA) with serum-free RPMI 1640, while culture medium supplemented with 20% fetal bovine serum (FBS) was added to the lower chamber. Cells in the Matrigel (Corning) were fixed with 4% paraformaldehyde, before being stained with 0.1% crystal violet (Beyotime, Shanghai, China), photographed, and counted.

### Western Blotting

Total proteins were extracted from human LUAD cells using the RIPA buffer (Beyotime), according to the manufacturer’s protocol. Protein concentration was determined using the BCA kit (Beyotime). Western blotting was performed to determine the expression levels of proteins involved in this study. Primary antibodies specific to N-cadherin and E-cadherin were purchased from Proteintech (Wuhan, China), while anti-vimentin antibody was purchased from Abcam (Cambridge, UK). The secondary antibodies were: 1:2000 goat anti-mouse IgG conjugated with HRP (Beyotime) and 1:2000 goat anti-rabbit IgG conjugated with HRP (Beyotime). The protein bands were imaged using an electrochemiluminescence (ECL) system, and the grey values were measured using by ImageJ software to evaluate relative protein levels normalized to β-actin expression level.

### Statistical Analysis

*NUF2* levels between LUAD and normal groups were compared using Student’s *t*-test. Pearson’s chi-square test was performed to assess the relationship between *NUF2* expression and clinical parameters. The overall survival (OS) and recurrence-free survival (RFS) differences between the low and high gene expression groups were compared by Kaplan–Meier analysis and log-rank test. Cox proportional hazards model was used to determine the independent prognostic factors related to OS or RFS. Prognostic factors screened by the Cox analysis of OS and RFS were incorporated into T stage, N stage, M stage, pathologic stage, primary therapeutic outcome, residual tumors, tumor status, and *NUF2* expression to construct the corresponding line graph. *NUF2* levels expression between LUAD and benign lung lesions were compared using the *t-*test function in R. Group differences in the biomarker levels were assessed with Mann–Whitney test or univariate general linear models, adjusted for age and sex as covariates. For all the analyses, results were considered significant at P < 0.05.

## Results

### Analysis of NUF2 Expression in Databases

**Figure 1:**
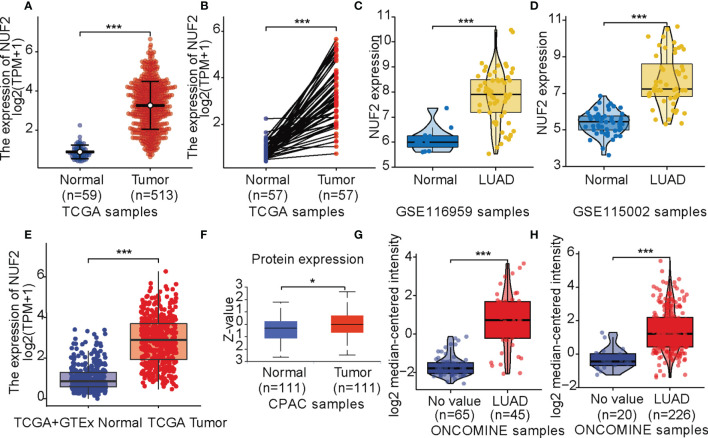
*NUF2* expression levels. **(A)** Expression levels of *NUF2* in lung adenocarcinoma (LUAD) and normal tissue from The Cancer Gene Atlas (TCGA). **(B)** the expression of *NUF2* in LUAD and its paired adjacent tissues. **(C)** mRNA expression of *NUF2* in LUAD was obtained from GSE116959. **(D)** mRNA expression of *NUF2* in LUAD was obtained from GSE115002. **(E)** Expression levels of *NUF2* in tumor and normal tissues in TCGA and Genotype-Tissue Expression data. **(F)** The protein expression of NUF2 in LUAD was obtained from the CPTAC dataset. **(G)** *NUF2* expression in tumor and normal tissues in LUAD from ONCOMINE database (Hou Lung). **(H)** *NUF2* expression in tumor and normal tissues in LUAD from ONCOMINE database (Okayama Lung). ***p < 0.001,*p < 0.05.

Data from TCGA revealed that *NUF2* was overexpressed in the LUAD samples relative to normal or para-cancerous samples (P < 0.001, **Figures 1A, B**). Based on GSE116959 and GSE115002 datasets, *NUF2* expression was remarkably higher than that in the normal lung specimens (P < 0.001, **Figures 1C, D**). We integrated normal tissue data into GTEx data. As shown in **Figure 1E**, *NUF2* mRNA levels also increased significantly in LUAD compared to those in their corresponding normal controls. Furthermore, we used UALCAN and ONCOMINE database to validate the expression of the NUF2 protein; our results validated the upregulation of NUF2 in LUAD samples (P < 0.001, **Figures 1F–H**) (14, 15).

### NUF2 Validation

**Figure 2:**
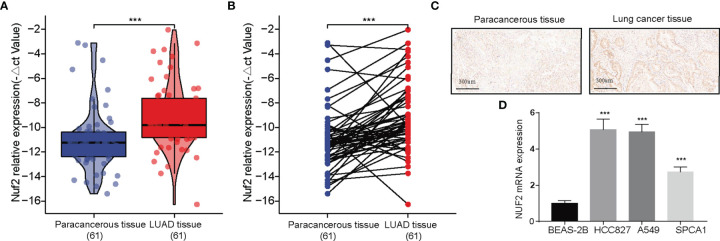
Expression analysis of *NUF2* in lung adenocarcinoma (LUAD). **(A, B)** Qualitative real-time PCR analysis of NUF2 in tumor and adjacent samples from our recruited cohort. **(C)** Immunohistochemical (IHC) staining sections for NUF2 of lung cancer tissues and cancer-adjacent normal lung tissues. **(D)** Differential expression of *NUF2* in in LUAD cell lines and normal lung epithelial cell line 2B. ***p < 0.001.

RT-qPCR assay and immunohistochemistry were was performed to assess the expression of *NUF2* in LUAD tissues. The expression was elevated in the LUAD samples of our cohort (**Figures 2A, B**). As shown in **Figure 2C**, it was demonstrated that the expression of NUF2 in lung cancer tissues was significantly higher than that in the corresponding paired para-tumor tissues, and the subcellular localization of NUF2 protein was in the nucleus and cytoplasm. Furthermore, *NUF2* was found to be overexpressed in LUAD cell lines (**Figure 2D**). As shown in **Table 1**, the serum concentrations of neuron specific enolase (NSE) in the group expressing high levels of NUF2 were higher than those in the low NUF2 expression group.

**Table 1:** Relationship between *NUF2* expression and clinicopathological parameters in our cohort.

Characteristic	Low expression of NUF2	High expression of NUF2	p
n	27	27	
Sex, n (%)			0.585
male	11 (20.4%)	14 (25.9%)	
female	16 (29.6%)	13 (24.1%)	
Age/years (%)			0.577
≤65	18 (33.3%)	15 (27.8%)	
>65	9 (16.7%)	12 (22.2%)	
T (%)			1.000
T1	22 (40.7%)	22 (40.7%)	
T1+2+3	5 (9.3%)	5 (9.3%)	
N stage (%)			1.000
N0	24 (44.4%)	23 (42.6%)	
N1	3 (5.6%)	4 (7.4%)	
M stage (%)			1.000
M0	26 (48.1%)	27 (50%)	
M1	1 (1.9%)	0 (0%)	
Tumor size (%)			0.053
≤2cm	20 (37%)	12 (22.2%)	
>2	7 (13%)	15 (27.8%)	
CEA (μg/L)	2.3 (1.55, 4.05)	2.8 (1.85, 4.9)	0.494
CY211 (ng/ml)	2.2 (1.8, 3.4)	2.6 (1.8, 3.92)	0.368
SCCA (μg/L)	0.6 (0.5, 0.8)	0.8 (0.5, 1)	0.247
NSE (ng/ml)	11.1 (9.1, 12.6)	13.1 (10.53, 17.95)	0.049
ProGRP (ng/l)	43.94 ± 17.03	51.41 ± 19.89	0.177

### Significant Association of NUF2 With Clinicopathological Features

**Figure 3:**
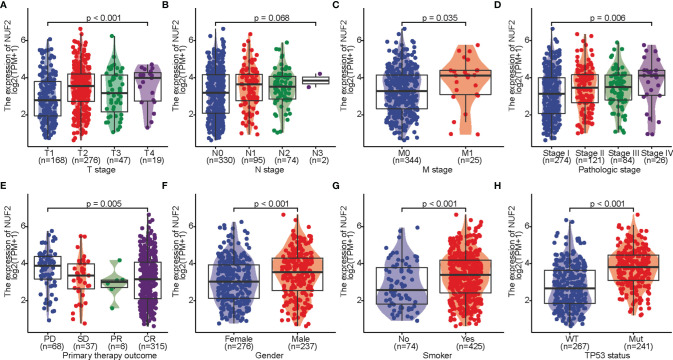
Correlation of *NUF2* expression of and clinicopathologic variables. **(A)** T stage, **(B)** N stage, **(C)** M stage. **(D)** pathologic stage. **(E)** primary therapy outcome, **(F)** sex, **(G)** smoking. **(H)** TP53 status.

**Figure 4:**
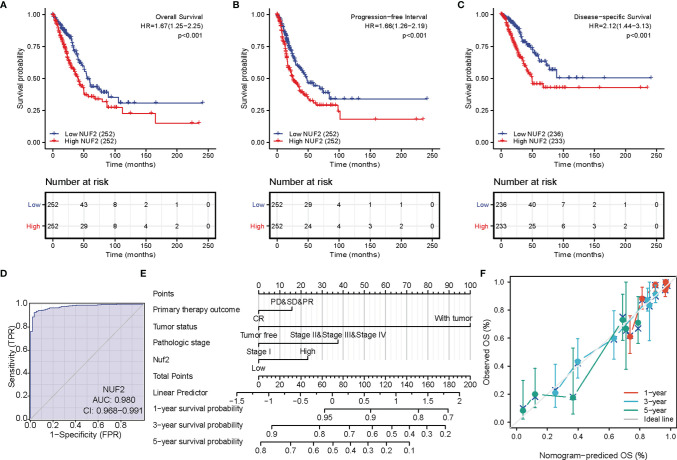
Prognostic value of *NUF2* expression in lung adenocarcinoma (LUAD). **(A)** Survival curves of overall survival. **(B)** Survival curves of progression-free interval. **(C)** Survival curves of disease-specific survival. **(D)** Receiver operating characteristic (ROC) curves of NUF2 in LUAD. **(E)** Prognostic nomogram that integrated NUF2 expression and other prognostic factors for OS in LUAD from The Cancer Genome Atlas data. **(F)** A calibration curve at 1-, 3- and 5-year.

The correlations between *NUF2* overexpression and its clinical relevance and prognostic value were analyzed by collating information from the LUAD-TCGA database. *NUF2* overexpression was significantly and positively correlated with a higher T stage (T2 Stage vs. T1 Stage, P < 0.001; T4 Stage vs. T1 Stage, P = 0.026), N stage (N1 Stage vs. N0 Stage, P = 0.048; T2 Stage vs. N0 Stage, P = 0.048), M stage (M1 Stage vs. N0 Stage, P = 0.035), pathological stage (Stage II vs. Stage I, P = 0.026; Stage III vs. Stage I, P = 0.017; Stage IV vs. Stage I, P = 0.018), primary therapeutic outcome (PD vs. CR, P < 0.001), sex (Male vs. Female, P < 0.001), smoking status (Yes vs. No, P < 0.001), and TP53 status (Mut vs. WT, P < 0.001) (**Table 2** and **Figures 3A–H**). Logistic regression analysis showed that *NUF2* overexpression was observably positively correlated with multiple factors, such as T stage (P < 0.001), N stage (P = 0.004), M stage (P = 0.039), pathologic stage (P = 0.001), and primary therapy outcome (P = 0.002), TP53 status (P < 0.001), Sex (P < 0.001), number of pack years smoked (P = 0.007) (**Table 3**). As shown in **Figures 4A–C**, survival analysis indicated that *NUF2* overexpression led to a significant reduction in OS (HR = 1.67, P < 0.001), PFI (HR = 1.66, P < 0.001), and DSS (HR = 2.12, P < 0.001). In addition, area under the curve (AUC) was 0.98, providing evidence of the favorable diagnostic ability of *NUF2* for LUAD (**Figure 4D**). Univariate Cox analysis revealed *NUF2* (HR = 1.674, P < 0.001) as a high-risk factor for LUAD (**Table 3**); meanwhile, multivariate Cox analysis highlighted that *NUF2* expression (HR = 1.839, P = 0.032) was independently related to OS (**Table 4**). In addition, based on the above results, we established a clinical nomogram for overall survival by fitting the expression of NUF2 and other clinical parameters. (**Figures 4E, F**) includes the calibration curves of our nomogram; plots were very close to the ideal line, which indicated the high predictive accuracy.

**Table 2:** Relationship between *NUF2* expression and clinicopathological parameters of LUAD.

Characters	Level	Low expression of NUF2	High expression of NUF2	p	Test
n		257	256		
T stage (%)	T1	104 (40.8%)	64 (25.1%)	0.001	
	T2	119 (46.7%)	157 (61.6%)		
	T3	25 (9.8%)	22 (8.6%)		
	T4	7 (2.7%)	12 (4.7%)		
N stage (%)	N0	178 (72.1%)	152 (59.8%)	0.016	exact
	N1	39 (15.8%)	56 (22.0%)		
	N2	30 (12.1%)	44 (17.3%)		
	N3	0 (0.0%)	2 (0.8%)		
M stage (%)	M0	172 (96.1%)	172 (90.5%)	0.055	
	M1	7 (3.9%)	18 (9.5%)		
Pathologic stage (%)	Stage I	155 (61.5%)	119 (47.0%)	0.007	exact
	Stage II	53 (21.0%)	68 (26.9%)		
	Stage III	36 (14.3%)	48 (19.0%)		
	Stage IV	8 (3.2%)	18 (7.1%)		
Tumor status (%)	Tumor free	164 (70.7%)	124 (54.9%)	0.001	
	With tumor	68 (29.3%)	102 (45.1%)		
Primary therapy outcome (%)	CR	174 (80.6%)	141 (67.1%)	<0.001	
	PD	19 (8.8%)	49 (23.3%)		
	PR	5 (2.3%)	1 (0.5%)		
	SD	18 (8.3%)	19 (9.0%)		
Sex (%)	Female	160 (62.3%)	116 (45.3%)	<0.001	
	Male	97 (37.7%)	140 (54.7%)		
Age (%)	<=65	102 (41.1%)	136 (55.3%)	0.002	
	>65	146 (58.9%)	110 (44.7%)		
	Peripheral Lung	59 (68.6%)	68 (66.0%)		
number pack years smoked (%)	<40	95 (57.2%)	79 (42.7%)	0.009	
	>=40	71 (42.8%)	106 (57.3%)		
Smoker (%)	No	49 (19.5%)	25 (10.1%)	0.004	
	Yes	202 (80.5%)	223 (89.9%)		
TP53 status (%)	Mut	78 (30.6%)	163 (64.4%)	<0.001	
	WT	177 (69.4%)	90 (35.6%)		

**Table 3:** Logistic regression analysis between *NUF2* expression and clinical features.

Characteristics	Odds Ratio in NUF2 expression	Odds Ratio (OR)	P value
T stage (T2&T3&T4 vs. T1)	510	2.06 (1.41-3.01)	<0.001
N stage (N1&N2&N3 vs. N0)	501	1.73 (1.19-2.52)	0.004
M stage (M1 vs. M0)	369	2.57 (1.09-6.76)	0.039
Pathologic stage (Stage II&Stage III&Stage IV vs. Stage I)	505	1.80 (1.26-2.57)	0.001
Primary therapy outcome (PD&SD&PR vs. CR)	426	2.03 (1.31-3.18)	0.002
Residual tumor (R1&R2 vs. R0)	361	0.83 (0.30-2.22)	0.706
Race (Asian&Black or African American vs. White)	446	1.61 (0.93-2.83)	0.094
TP53 status (Mut vs. WT)	508	4.11 (2.85-5.98)	<0.001
Sex (Male vs. Female)	513	1.99 (1.40-2.84)	<0.001
number pack years smoked (>=40 vs. <40)	351	1.80 (1.18-2.75)	0.007

**Table 4:** Univariate and multivariate Cox model.

Characteristics	Total (N)	HR (95% CI) Univariate analysis	P value Univariate analysis	HR (95% CI) Multivariate analysis	P value Multivariate analysis
T stage (T2&T3&T4 vs. T1)	501	1.668 (1.184-2.349)	0.003	1.372 (0.712-2.645)	0.344
N stage (N1&N2&N3 vs. N0)	492	2.606 (1.939-3.503)	<0.001	1.565 (0.679-3.610)	0.293
M stage (M1 vs. M0)	360	2.111 (1.232-3.616)	0.007	1.021 (0.377-2.764)	0.967
Pathologic stage (Stage II&Stage III&Stage IV vs. Stage I)	496	2.975 (2.188-4.045)	<0.001	0.778 (0.316-1.914)	0.584
Primary therapy outcome (PD&SD&PR vs. CR)	419	2.818 (2.004-3.963)	<0.001	2.457 (1.423-4.243)	0.001
Residual tumor (R1&R2 vs. R0)	352	3.973 (2.217-7.120)	<0.001	1.976 (0.732-5.334)	0.179
Tumor status (With tumor vs. Tumor free)	450	6.211 (4.258-9.059)	<0.001	5.843 (3.229-10.576)	<0.001
Sex (Male vs. Female)	504	1.060 (0.792-1.418)	0.694		
Age (>65 vs. <=65)	494	1.228 (0.915-1.649)	0.171		
Race (White vs. Asian&Black or African American)	446	1.422 (0.869-2.327)	0.162		
Anatomic neoplasm subdivision (Right vs. Left)	490	1.024 (0.758-1.383)	0.878		
Anatomic neoplasm subdivision2 (Peripheral Lung vs. Central Lung)	182	0.913 (0.570-1.463)	0.706		
number pack years smoked (>=40 vs. <40)	345	1.038 (0.723-1.490)	0.840		
Smoker (Yes vs. No)	490	0.887 (0.587-1.339)	0.568		
TP53 status (Mut vs. WT)	499	1.254 (0.936-1.680)	0.130		
KRAS status (Mut vs. WT)	499	1.087 (0.779-1.517)	0.623		
NUF2 (High vs. Low)	504	1.674 (1.246-2.250)	<0.001	1.839 (1.055-3.204)	0.0 32

### Interaction Network of NUF2

**Figure 5:**
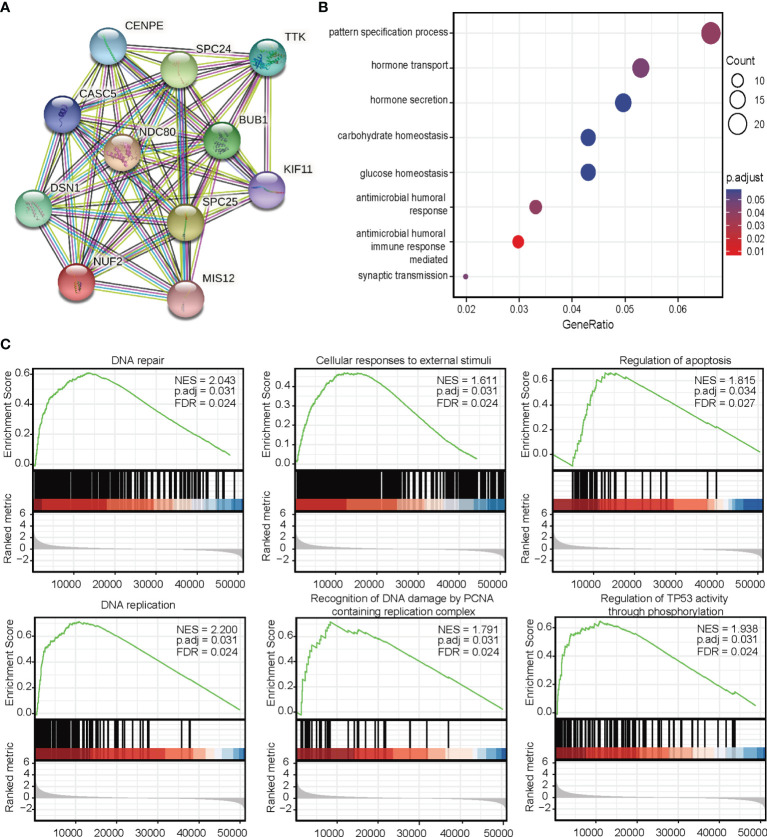
**(A)** Protein-protein interaction network analysis of NUF2-related genes. **(B)** Gene Ontology analysis. **(C)** Gene set expression analysis.

Next, we investigated the associations between *NUF2* and known and predicted proteins. As shown in **Figure 5A**, the top 10 predicted partners with their scores were: *BUB1* (0.994), *CASC5* (0.988), *CENPE* (0.988), *DSN1* (0.992), *KIF11* (0.987), *MIS12* (0.983), *NDC80* (0.999), *SPC24* (0.999), *SPC25*(0.998), and *TTK* (0.991).

### Enrichment Analyses

To predict the function of *NUF2*, we performed a GO analysis. Functional enrichment analysis of genes in this network showed that they were enriched for immune response (**Figure 5B**). GSEA indicated an enrichment in DNA repair, cellular responses to external stimuli, regulation of apoptosis, DNA replication, recognition of DNA damage by the PCNA containing replication complex, and regulation of TP53 activity through phosphorylation pathways (**Figure 5C**).

### Relevance of NUF2 Expression in Immune Infiltration

**Figure 6:**
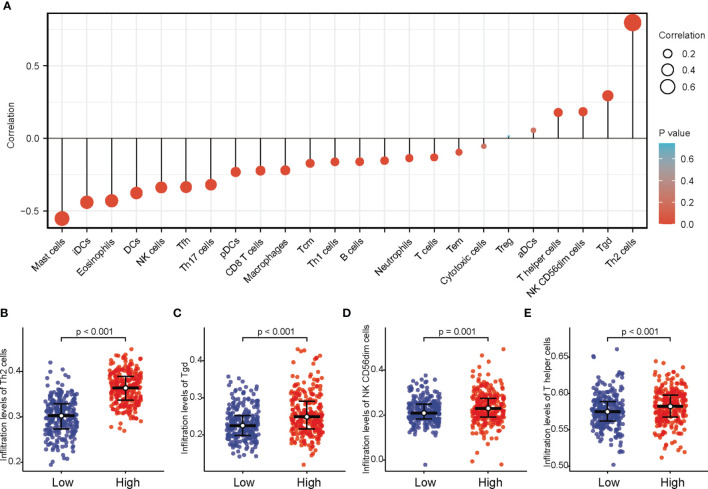
Association between the expression level of *NUF2* and immune infiltration in the tumor microenvironment. **(A)** The forest plot shows the correlation between *NUF2* expression level and 24 immune cells. **(B)** the enrichment scores of *NUF2* expression in Th2 cells and macrophages; **(C)** Enrichment scores of *NUF2* expression in Tgd cells; **(D)** Enrichment scores of *NUF2* expression in NK CD56dim cells. **(E)** Enrichment scores of *NUF2* expression in T helper cells.

Correlations between *NUF2* overexpression and immune cell infiltration level in LUAD tissues were evaluated using the ssGSEA database. The analysis revealed remarkable positive correlation of *NUF2* overexpression with Th2 cells, T gamma delta (Tgd), NK CD56dim cells, and T helper cells in LUAD (**Figure 6**).

### NUF2 Promotes Cell Proliferation, Invasion, and Migration in LUAD

**Figure 7:**
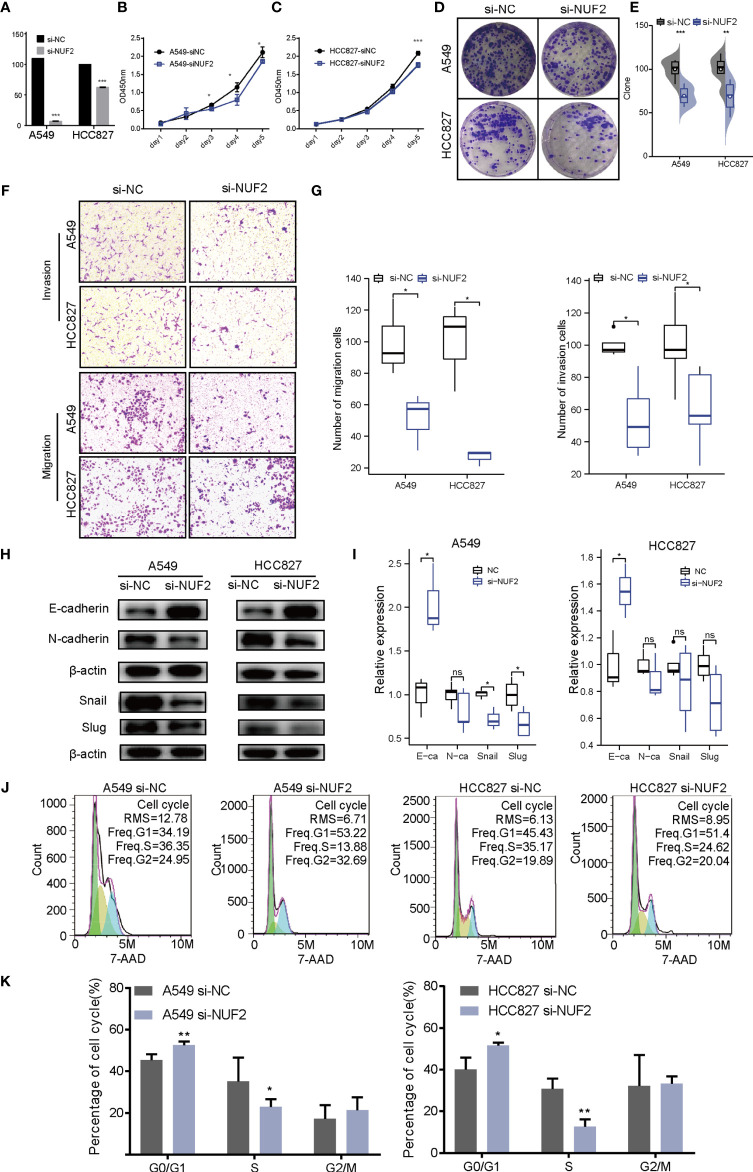
Biological function of *NUF2* in lung adenocarcinoma (LUAD) cells. **(A)** Reverse transcription-quantitative polymerase chain reaction analysis of *NUF2* in A549 and HCC827 cells transfected with si-NC or si-NUF2. **(B, C)** Cell proliferation rates as determined by CCK8 assays in A549 and HCC827 cells transfected with control si-NC or si-NUF2. **(D, E)** Colony formation assay for evaluating the clonogenic ability of A549 and HCC827 cells. **(F, G)** The invasive ability of A549 and HCC827 cells was detected by transwell assay. **(H, I)** Expression of epithelial-mesenchymal transition markers in NUF2-silenced samples as measured using western blotting assays. **(J, K)** Effect of NUF2 knockdown on cell cycle progression. ***p < 0.001,**p < 0.01,*p < 0.05.

To verify the effect of *NUF2* on the biological function of LUAD cells, siRNA targeting *NUF2* (si- *NUF2*) was employed to knock down the expression of *NUF2* in HCC827 and A549 cell lines. RT-qPCR results indicated that si-*NUF2* effectively inhibited *NUF2* expression in LUAD cell lines (**Figure 7A**). As shown in **Figures 7B–E**, downregulation of *NUF2* expression significantly inhibited cell proliferation. Furthermore, it dramatically reduced the invasion and migration capacities of LUAD cells (**Figures 7F, G**). Epithelial-mesenchymal transition (EMT) is characterized by the upregulated expression of N-cadherin followed by the downregulated expression of E-cadherin. *NUF2* silencing increased E-cadherin expression and decreased that of N-cadherin (**Figures 7H, I**). These findings indicated that *NUF2* may promote EMT. Cell cycle analysis revealed that *NUF2* induced cell cycle arrest at G0/G1 (**Figures 7J, K**). Collectively, these findings suggested that *NUF2* may drive tumor progression and act as an oncogene.

## Discussion

Molecular biomarkers can guide the diagnosis, prognosis, and treatment of patients with LUAD. In combination with other genes, *NUF2* has been reported to be a biomarker of LUAD, which is consistent with our results. *NUF2* overexpression can increase the proliferative ability of liver (16), pancreatic (17), and breast cancer cells (18, 19). Previous studies have analyzed *NUF2* in lung adenocarcinoma using multiple omics methods, verifying that *NUF2* mRNA is highly expressed in lung adenocarcinoma cell lines. However, the potential biomarkers and important functional genes have not been tested in clinical cohorts and cell function experiments in LUAD. In this study, we performed a more systematic clinical correlation analysis of *NUF2*. Based on the results of univariate and multivariate Cox analyses, *NUF2* was indicated as a high-risk factor for LUAD development, and it could serve as an independent indicator to predict the clinical outcomes of patients with LUAD. Chen et al. analyzed by GO, KEGG, and GSEA enrichment and found that *NUF2* is significantly enriched in the cell cycle, especially during DNA replication (8). Previous studies have also found that knockdown of *NUF2* induces cell cycle arrest in pancreatic cancer and breast cancer (17, 18). However, in our research, we found that *NUF2* has a minimal effect on cell cycle. The differential effect of *NUF2* on the cycle may indicate that the *NUF2* gene has various effects on epigenetic regulation in different cancer types. Notably, downregulated *NUF2* expression was associated with decreased cell proliferation. In addition, the results demonstrated *NUF2* to be involved in regulating EMT, which has not been previously reported. Collectively, these results provide direct evidence that *NUF2* acts as an oncogene in LUAD.

GSEA results showed that *NUF2* is involved in proliferation and apoptosis-related pathways, including proliferating cell nuclear antigen (*PCNA*) and *TP53* pathways. PCNA is related to tumor growth rate; therefore, PCNA expression is used as an important proliferative marker (20). *TP53* act as a tumor suppressor gene by regulating apoptosis and the cell cycle as well as mediating DNA damage repair (21). Therefore, we hypothesized that the functions of *NUF2* in tumorigenesis are mediated by the PCNA and P53 pathways. By establishing a PPI network, we further identified that these genes play a key role in cell cycle-related meiosis. The PPI was positively associated with top 10 *NUF2* co-expressed genes (*BUB1*, *CASC5*, *CENPE*, *DSN1*, *KIF11*, *MIS12*, *NDC80*, *SPC24*, *SPC25*, and *TTK*). Chen et al. had reported that the increased expression levels of *BUB1B* and *BUB1* was related to the OS in patients with LUAD (22). Incidentally, *CENPE* encodes centromere-associated protein E, which is a human kinetochore protein that promotes lung adenocarcinoma proliferation (23).

Immune cells present in the tumor-microenvironment play a key role in tumor tissues, with increasing evidence supporting their clinicopathological significance in predicting the survival status of and therapeutic efficacy in cancer patients (24, 25). According to a recent report, various immune cells subtypes, such as Th2, Tgd, NK CD56dim cells, and T helper cells, are vital components of the tumor microenvironment (26). Moreover, studies have reported that Th2 cell infiltration correlates with reduced survival in patients with pancreatic cancer and clear cell renal cell carcinoma. Furthermore, in our study, the Th2 cell infiltration level was significantly higher in the high NUF2 expression group; therefore, we hypothesized that the infiltration level of Th2 accelerates the progression of LUAD.

Despite presenting some credible data, our study did have some limitations. Firstly, it had some inherent limitations due to its retrospective design and small sample size. To further confirm our results, a large-scale prospective study would be required. Secondly, although the report indicated the biological effects related to EMT, the study failed to explore the underlying mechanism of the signaling pathways involving *NUF2*. Thus, further studies are required to investigate the mechanism responsible for regulating NUF2 expression and its role in LUAD.

In conclusion, to the best of our knowledge, this is the first comprehensive analysis of the expression pattern and clinical significance of *NUF2* in LUAD. Our results revealed that *NUF2* is overexpressed in LUAD compared to neighboring tissues, and that *NUF2* expression is an independent prognostic factor related to OS. Overall, the study provides new evidence of *NUF2* being closely linked with the development and progression of LUAD.

## Data Availability Statement

The datasets presented in this study can be found in online repositories. The names of the repository/repositories and accession number(s) can be found in the article/**Supplementary Material**.

## Ethics Statement

The studies involving human participants were reviewed and approved by This study was approved by the Institutional Ethical Review Committee of the First Affiliated Hospital of Wenzhou Medical University (YS2018001). The patients/participants provided their written informed consent to participate in this study.

## Author Contributions

FJ conceived and designed the experiments, performed the experiments, analyzed the data, prepared figures and/or tables, authored or reviewed drafts of the paper, and approved the final draft. XH and XY performed the experiments, analyzed the data, prepared figures and/or tables, and approved the final draft. HZ analyzed the data, prepared figures and/or tables. YW conceived and designed the experiments, authored or reviewed drafts of the paper, and approved the final draft. All authors contributed to the article and approved the submitted version.

## Funding

Zhejiang Provincial Research Center for Cancer Intelligent Diagnosis and Molecular Technology (JBZX-202003). Wenzhou Municipal Science and Technology Bureau of China (Y20180113). Zhejiang Provincial Natural Science Foundation (LQ22H200005). Scientific Research Incubation Project of The First Affiliated Hospital of Wenzhou Medical University (FHY2019084).

## Conflict of Interest

The authors declare that the research was conducted in the absence of any commercial or financial relationships that could be construed as a potential conflict of interest.

## Publisher’s Note

All claims expressed in this article are solely those of the authors and do not necessarily represent those of their affiliated organizations, or those of the publisher, the editors and the reviewers. Any product that may be evaluated in this article, or claim that may be made by its manufacturer, is not guaranteed or endorsed by the publisher.
