# Supplementary figures and images for: NUF2 Expression Promotes Lung Adenocarcinoma Progression and Is Associated With Poor Prognosis
Source: Front Oncol. 2022 Jun 23;12:795971. doi: 10.3389/fonc.2022.795971 (PMC9259841; doi:10.3389/fonc.2022.795971)

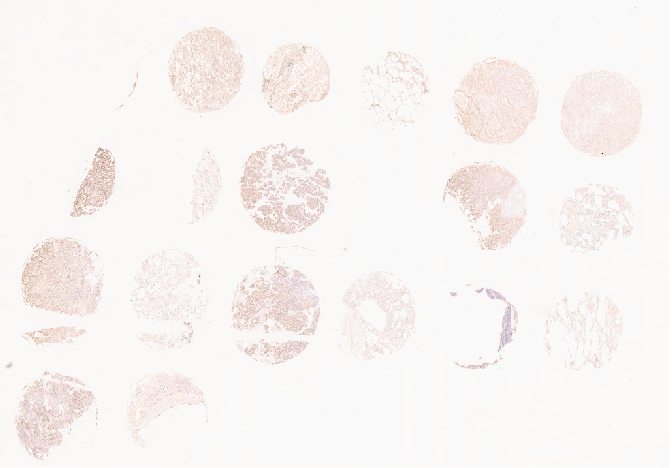

Supplement: Supplementary file 1 [file Image_1.tif]
